# Nano-liter perfusion microfluidic device made entirely by two-photon polymerization for dynamic cell culture with easy cell recovery

**DOI:** 10.1038/s41598-023-27660-x

**Published:** 2023-01-11

**Authors:** Hanna J. McLennan, Adam J. Blanch, Samuel J. Wallace, Lesley J. Ritter, Shauna L. Heinrich, David K. Gardner, Kylie R. Dunning, Marty J. Gauvin, Allison K. Love, Jeremy G. Thompson

**Affiliations:** 1 Fertilis Pty Ltd, The University of Adelaide, Frome Road, Helen Mayo South, Adelaide, SA 5005 Australia; 2 Virtual Ark Pty Ltd, 73 Woolnough Road, Semaphore, SA 5019 Australia; 3 School of Biomedicine, Faculty of Health and Medical Sciences, The University of Adelaide, Adelaide, SA 5005 Australia; 4 Melbourne IVF, East Melbourne, VIC 3002 Australia; 5 School of BioSciences, University of Melbourne, Parkville, VIC 3010 Australia; 6 Robinson Research Institute, Adelaide Medical School, The University of Adelaide, Adelaide, SA 5005 Australia; 7 Australian Research Council Centre of Excellence for Nanoscale BioPhotonics, The University of Adelaide, Adelaide, SA 5005 Australia; 8 Institute for Photonics and Advanced Sensing, The University of Adelaide, Adelaide, SA 5005 Australia; 9 R&D Certainty Pty Ltd, 73 Woolnough Road, Semaphore, SA 5019 Australia; 10 ART Lab Solutions Pty Ltd, 10 Pulteney Street, Adelaide, SA 5005 Australia

**Keywords:** Embryology, Cellular imaging

## Abstract

Polydimethylsiloxane (PDMS) has been the material of choice for microfluidic applications in cell biology for many years, with recent advances encompassing nano-scaffolds and surface modifications to enhance cell-surface interactions at nano-scale. However, PDMS has not previously been amenable to applications which require complex geometries in three dimensions for cell culture device fabrication in the absence of additional components. Further, PDMS microfluidic devices have limited capacity for cell retrieval following culture without severely compromising cell health. This study presents a designed and entirely 3D-printed microfluidic chip (8.8 mm × 8.2 mm × 3.6 mm) using two-photon polymerization (2PP). The ‘nest’ chip is composed of ten channels that deliver sub-microliter volume flowrates (to ~ 600 nL/min per channel) to 10 individual retrievable cell sample ‘cradles’ that interlock with the nest to create the microfluidic device. Computational fluid dynamics modelling predicted medium flow in the device, which was accurately validated by real-time microbead tracking. Functional capability of the device was assessed, and demonstrated the capability to deliver culture medium, dyes, and biological molecules to support cell growth, staining and cell phenotype changes, respectively. Therefore, 2PP 3D-printing provides the precision needed for nanoliter fluidic devices constructed from multiple interlocking parts for cell culture application.

## Introduction

In vitro cell culture is a critical technique to interrogate the processes of biological systems. While this technique has advanced every area of medical research, it has significant limitations by creating a static environment thereby inducing unstirred layers that contribute to compromised cell function^1^. In comparison, the in vivo environment is dynamic, where blood delivers nutrients and removes wastes from tissues using fluid movement. This critical difference contributes to the failure to translate cell culture research breakthroughs to medical treatments. Indeed, 90% of candidate drugs with therapeutic promise observed through cell culture experimentation fail to translate benefit in clinical trials^2^. Therefore, introducing dynamic conditions to traditional cell culture practices is critical to improve how accurately in vivo biological conditions are replicated in cell culture models.

Microfluidic devices have been developed over several decades to enable the dynamic delivery of fluids during in vitro cell culture. Current microfluidic devices display wide ranges of geometry, flow rates and mechanical capabilities^3–5^. For cell culture, these devices have significant promise as a discovery and diagnostic tool^6–9^, where cell behavioral differences in response to treatment are measured within the device. Under these conditions, measurable cellular responses are determined mostly by microscopy, especially fluorescence microscopy, conducted within the device itself. However, a limitation is the inability to harvest cells easily to assess other molecular readouts, such as genomic expression of cells, or performing multiple analyses within these devices, particularly for 3D cell culture applications. As such, the impact of microfluidics has been restricted when compared to other more accessible in vitro cell culture systems^10^.

Current microfluidic chips are typically molded from polydimethylsiloxane (PDMS) on a glass substrate due to its transparency, biocompatibility and gas permeability^11^. More recently, PDMS nano-structure and nano-scaffold devices comprising multiple components have been fabricated to improve cell-substrate interactions^12,13^, including via laser lithography^14^ and micro reactive ion etching^15^, however to date its use to construct chips with nanoscale directional geometries for cell culture has been limited. Further, water absorption and evaporation of the small media volumes present in PDMS channels leads to unphysiological increases in medium osmolality, initiating cell shrinkage and death^12,13^. Therefore, a manufacturing technique to create micron-sized features that minimizes evaporation and allows for complex geometry in three dimensions is required to advance the use of microfluidics for cell culture applications. Additionally, the ability to retrieve cells after culture through the removal of parts of the device would facilitate a wider range of post-culture applications.

A technology capable of addressing these challenges is three-dimensional (3D) microprinting using two photon polymerization (2PP), also referred to as ‘direct laser writing’. 2PP printing is a high-resolution micro-additive manufacturing technique using photosensitive material, based on the optical process of two photon absorption (2PA)^16^. In 2PP printing, a high-power focused laser creates a nonlinear energy distribution centered at the laser focal point, whereby 2PA excitation induces monomer crosslinking of the material with polymerization limited to the voxel around the focal point, creating a three-dimensional nanostructure^16^. Consequently, unlike layer-by-layer moulding techniques utilized in PDMS microfluidic devices, 2PP can fabricate precise sub-micron structures with high resolution features, smaller than the wavelength of the laser designed to suit the geometry of the sample, optimize microfluidic dynamics within the device, and enable sample retrieval^16,17^. Importantly, 2PP supports the use of photopolymers that are biocompatible and non-cytotoxic for cell culture applications^18^. However, the application of 2PP within cell culture has largely focused on printing micron- and sub-micron sized scaffolds for 3D cell culture^19^. More recently, studies have focused on the incorporation of specific 2PP printed features within other cell culture fluidic materials^20,21^. Nevertheless, a major limitation of 2PP has been the size of print achievable^22^ and, as such, this technology has not previously been used to print a 2PP flow-through microfluidic chip any larger than 1 mm × 1 mm × 0.01 mm^23,24^. Recent advancements in 2PP technology have increased the print size achievable, with the NanoOne high-resolution 3D printer supporting an accessible writing area of 100 mm × 120 mm^25^. This advancement could widen the applications of 2PP in many fields.

**Figure 1:**
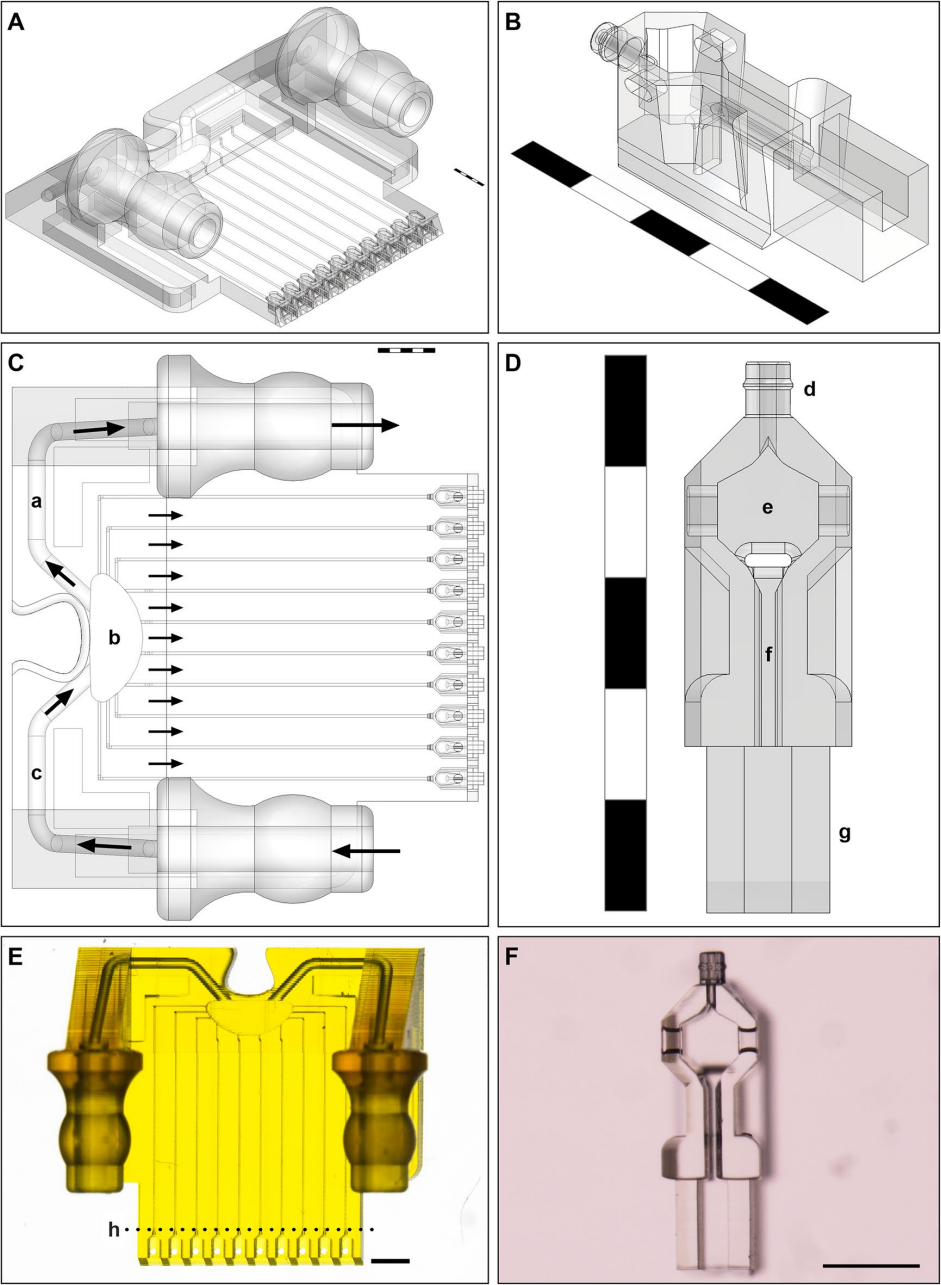
Computer aided design (CAD) models of the nest and cradle (Scale bars **A**–**E** = 1 mm; **F** = 300 µm). (**A**) Isometric view of nest; (**B**) Isometric view of cradle; (**C**) Top view of nest (**a**—outlet channel, **b**—back reservoir, **c**—inlet channel, arrows indicate direction of fluid flow); (**D**) Top view of cradle (**d**—nozzle, **e**—cell chamber which is open at the top to enable cell manipulation, **f**—injection channel, **g**—fin); (**E**) Top image of nest at 1 × magnification on Nikon SMZ18 stereomicroscope. The dimensions of the nest ‘chip’ are 8.8 mm × 8.2 mm × 3.6 mm (nest printed at 10 × in adaptive resolution in course mode above line **h** and fine mode below line **h**); (**F**) Top image of cradle at 8 × magnification with a Nikon SMZ18 stereomicroscope (cradles printed at 20 × in course infill mode).

**Figure 2:**
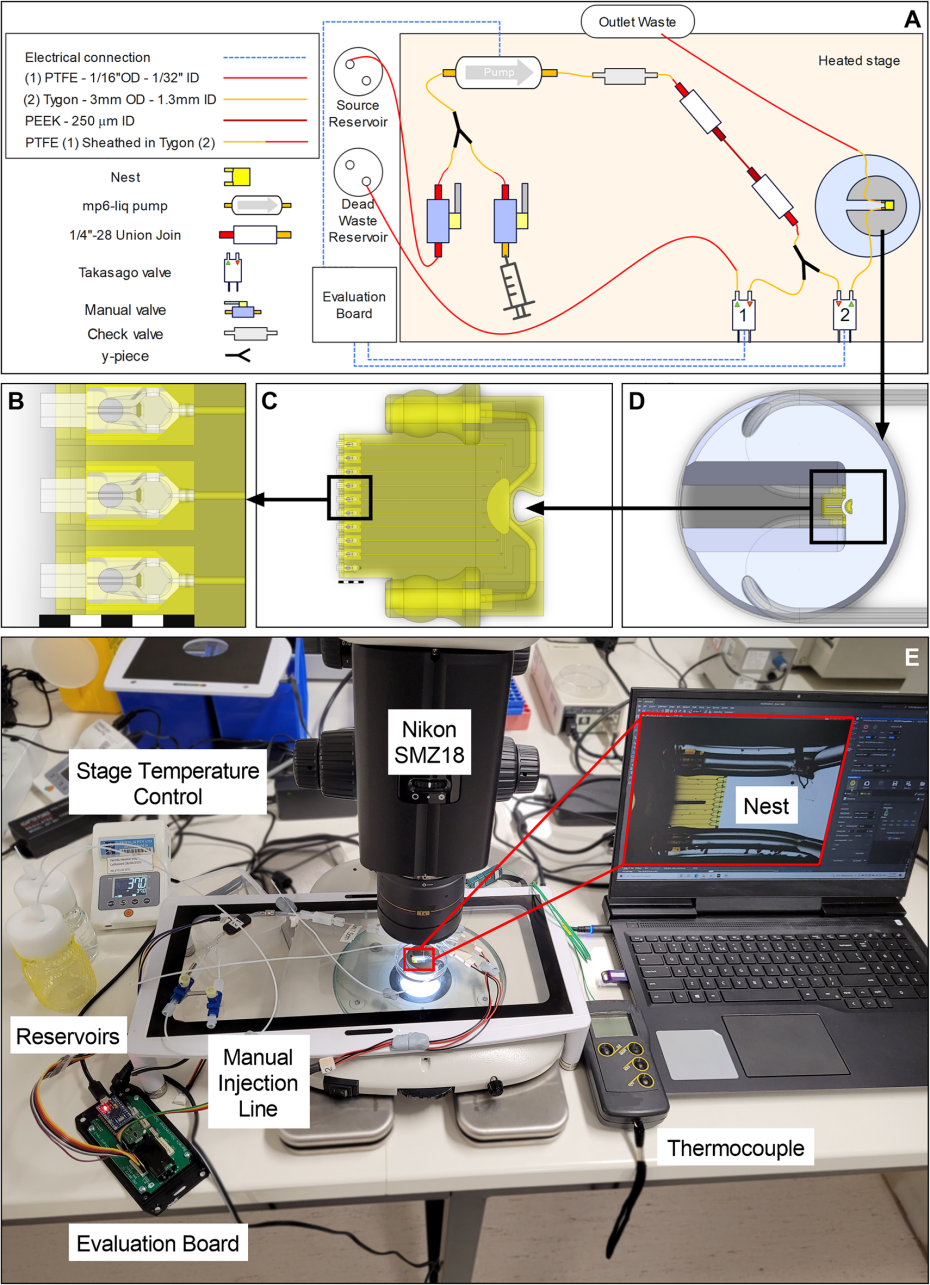
Microfluidic testbed for nest-cradle device (Scale bars = 1 mm). (**A**) Diagrammatic representation of testbed components (components and positions are not to scale); (**B**) Zoom in on model of three central cradles inserted into the nest, which acts as the roof with cell loading holes for each cradle; (**C**) Zoom in on model of the whole nest with ten cradles inserted; (**D**) Model of nest with tubing attached secured by titanium holder in 60 mm petri dish; (**E**) Photographic image of testbed set up in the laboratory with a Nikon SMZ18 stereomicroscope. An Olympus IX83 inverted microscope was used in place of the SMZ18 for particle tracking velocimetry.

This paper presents an entirely 2PP printed sub-microfluidic device 8.8 mm × 8.2 mm × 3.6 mm in size, with the aim to validate its use for 3D cell culture. The device is designed to deliver regulated low flow rates through 10 channels to 10 independent interlocking 2PP cell culture vessels, whilst simultaneously enabling the retrieval of cells at the conclusion of culture. The computer-aided designed (CAD) model is presented (Fig. 1), which was 2PP printed and attached to a system of pumps and tubing referred to here as the testbed to deliver fluids through the system (Fig. 2), along with fluidic modelling predicting the flow rates that can be achieved utilizing this microstructure and validation of flow rate via microbead tracking. Three independent biological outcome measures demonstrate cell growth, and the delivery of stains and hormones to alter cell appearance and phenotype with retrieval of the cells occurring at the conclusion of culture. The potential future capabilities and applications of this system are then discussed.

## Results

### Computational fluid dynamic analysis of design

**Figure 3:**
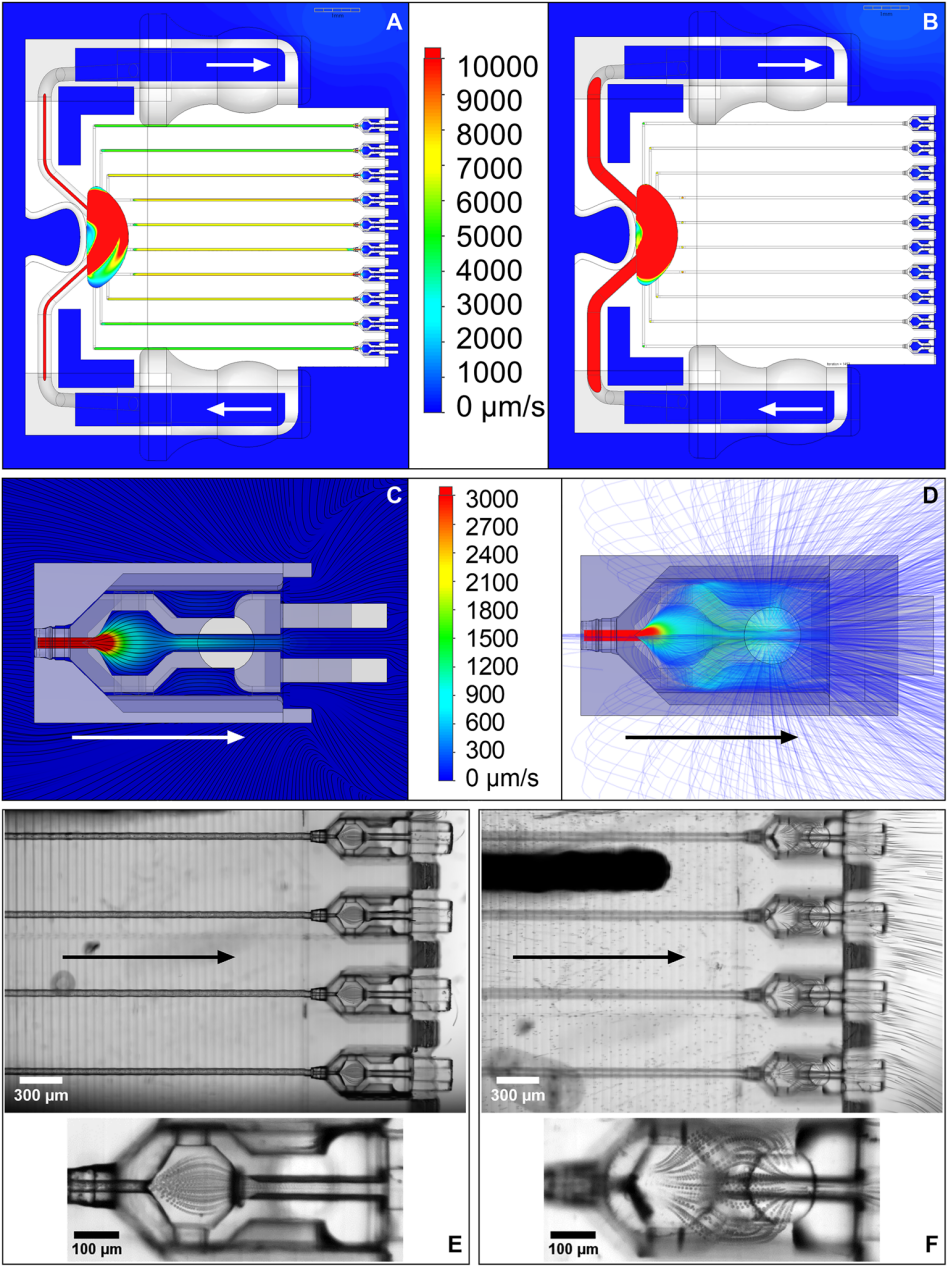
Predicted and actual flow patterns through the nest-cradle device (Scale bars **A**–**B** = 1 mm; **C**–**D** cradle length from nozzle to fin = 1 mm; **E**–**F** = as labelled; arrows indicate direction of fluid flow). (**A**) Computational fluid dynamic (CFD) modelling showing distribution of flow across the nest channels; (**B**) CFD modelling at nest surface showing flow through the inlet, back reservoir, and outlet located 250 µm above the centre of the channels; (**C**) CFD modelling showing the smoothing effect of the nozzle within an individual cradle; (**D**) CFD modelling of the flow trajectories within an individual cradle from below; (**E**) Projection of bead tracks from above with the focal plane set at the center of the channels; (**F**) Projection of bead tracks from above with the focal plane set to the nest top surface.

Computational Fluid Dynamic (CFD) analysis demonstrated that medium flow across each of the channels had a similar velocity (Fig. 3A, B), with flow measured at 7000 μm/s and the nozzle having a regulating effect on the flow (Fig. 3C, D). As the nozzle constricts the channel down to 30 µm diameter, the flow velocity increased and equalized to create a consistent flow rate into each of the cradles. Upon exiting the nozzle, fluid slows down, such that by the time it has crossed the cell chamber it has reduced significantly to ~ 400 μm/s. This reduction in velocity was the same across each of the channels. However, flow at the top of the cradle cavities was approximately 30% higher in the central channels compared to the outer ones as the flow became more dispersed (Fig. 3B).

### Demonstration and calculation of flow rate to replicate CFD

**Figure 4:**
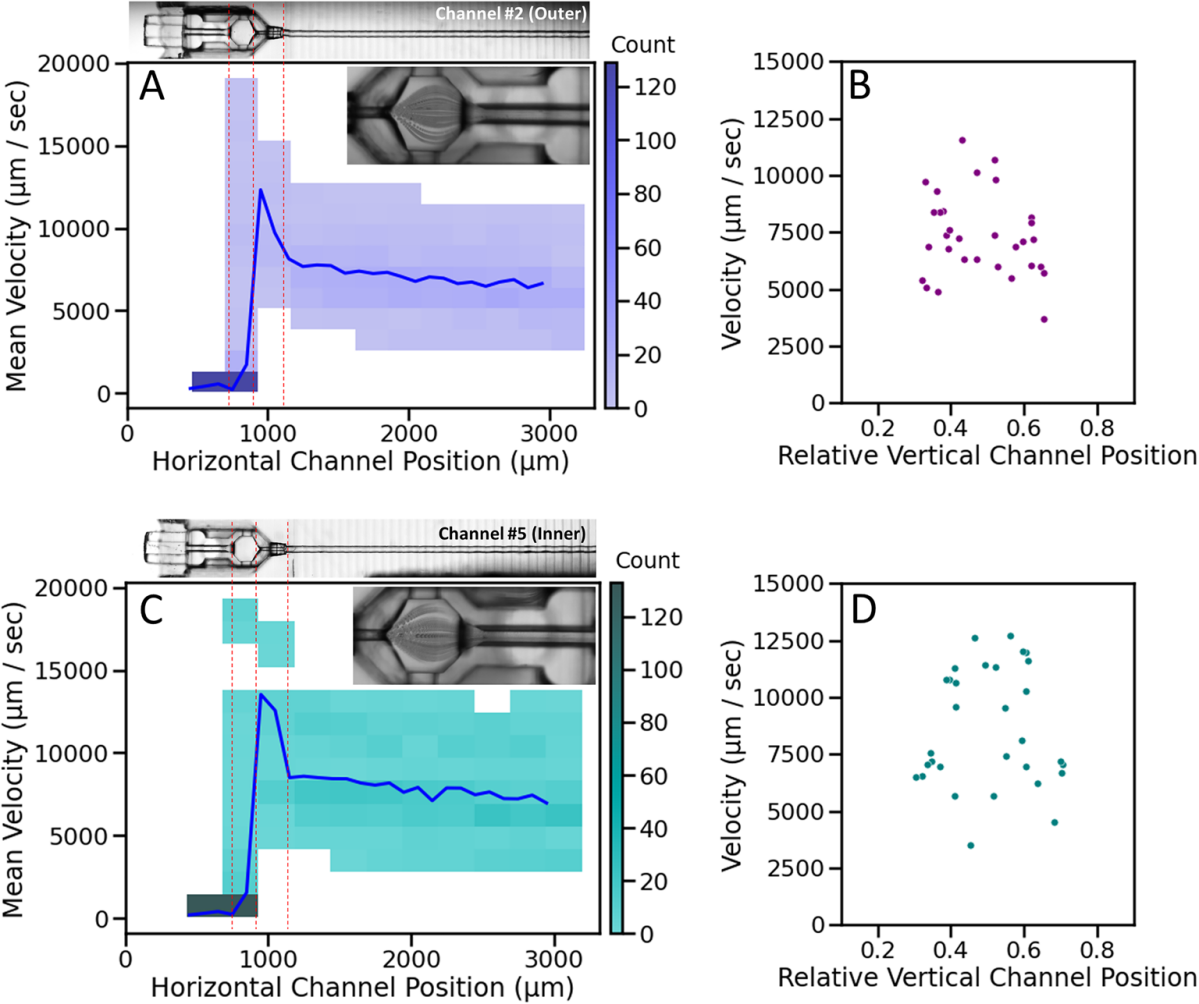
2D histograms of bead velocity per channel length, for the second-to-outermost channel #2 (**A**) and innermost channel #5 (**C**). The blue trace is the mean velocity profile, with the corresponding projection of bead tracks (inset). Velocity profiles are shown within the channel for channel #2 (**B**) and #5 (**D**), relative to the channel walls.

Particle velocimetry was performed to validate the results of CFD, where microbeads of ~ 6–7 μm diameter were flowed through the device and recorded at a high frame rate. The positions of the beads were projected over time, revealing flow patterns similar to predictions from the CFD analysis (Fig. 3E–F). Using high-speed video, the displacement of individual particles was tracked from frame to frame within the separate channels, and their respective velocities at each point were determined using the known time between frames. Mean particle velocities (n ≥ 30) for the equal innermost channel (#5, Fig. 4A, B) and second-to-outermost channel (#2, Fig. 4C, D) are plotted as a function of position in the horizontal imaging plane (Fig. 4A, C).

**Figure 5:**
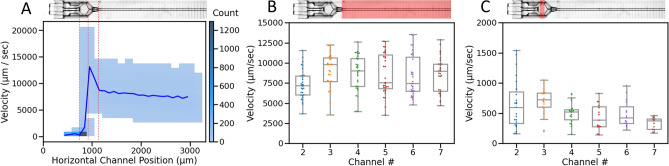
Bead velocity distributions captured at 4 × magnification in a single field of view for 6 channels (positions 2–7); (**A**) mean velocities combined from all channels overlaid on a 2D histogram of counts binned by velocity and position; with mean velocities per bead, per channel, calculated along the straight section (**B**) and within the cell chamber (**C**).

The velocity of the particles increased slightly as they moved along the channel (from a mean of 7501 to 8665 μm/s), peaking at 13,132 μm/s with rapid acceleration as the particles passed through the nozzle restriction. The particles then decelerated rapidly (with a mean of 1815 μm/s) as they entered the cell chamber as predicted by CFD (Fig. 5A). Some particles traversed the chamber and passed through the injection channel, while others egressed through the hole in the top of the nest as the modelling predicted (Fig. 3D, F). The distribution of mean particle velocities in the vertical imaging plane (Fig. 4B, D) revealed greater velocities in the channel centre compared to along the channel edges, which is consistent with the expectations of laminar flow in channels of these dimensions.

The velocity profile measured within each channel was similar, and the velocities of at least 30 particles per channel, for each of the 6 channels within a single field of view at 4 × magnification, were analysed over the same 4.3 s time window, comprising 270 video frames, and pooled (Fig. 5A). Noting that mean velocities were calculated per bead to avoid bias to lower velocities, as slower beads contributed more data points, the overall mean velocity from the straight section of the channels (highlighted area in Fig. 5B) was calculated to be 8465 ± 2253 μm/s, which corresponded to a flow rate of 1.00 ± 0.3 μL/min per channel. Uniformity of velocities between the channels was high for channels 3–7 (Fig. 5B; flow rates of 1.07, 1.03, 1.02, 0.99 and 1.01 μL/min respectively), with a reduction in particle velocities, and therefore calculated flow rate of 0.87 μL/min in channel 2. Channels 1 and 10 were unable to be assessed visually due to the nest’s titanium holder. However, from CFD results, flow in the outermost channels is expected to be further reduced. Inside the cell chamber, the particle velocities were significantly reduced, with a mean velocity for all channels measured as 523 ± 221 μm/s within the chamber centre (red highlighted area in Fig. 5C). The difference is flow velocity was lessened in the cradles compared to the channels since the volume of the former is much larger and thus any differences are greatly diminished. Particle velocity did not differ significantly within the cell chamber between the outer channel (2) and inner channels (5 and 6).

### Demonstration of medium delivery to the cradle

**Figure 6:**
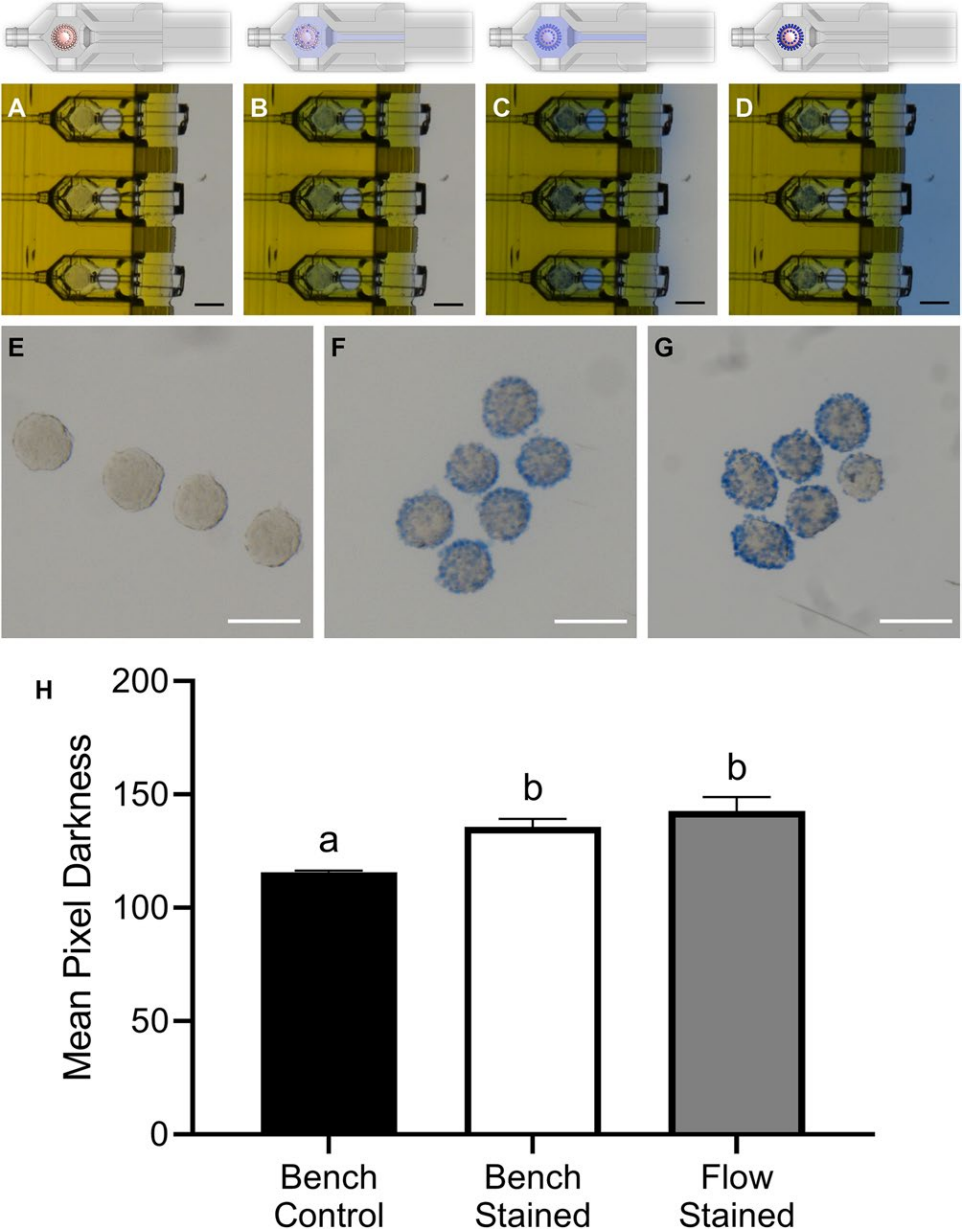
Permeabilized HEK293 cell spheroids treated with trypan blue using dynamic flow delivery (Scale bars = 200 µm; 1 rep, n = 6). (**A**) No staining, t = 1 min; (**B**) Staining in progress, t = 4 min; (**C**) Washing stain out, t = 10 min; (**D**) Staining and washing complete, t = 17 min; (**E**) Control untreated spheroids; (**F**) Spheroids stained in standard static conditions on the bench; (**G**) Spheroids stained by delivering the trypan blue stain through the fluidic device as shown in **A**–**D**; (**H**) Mean pixel darkness (± SD) of greyscale images **E**–**G** of the area in the images containing spheroids (background excluded); Brown-Forsythe and Welch’s ANOVA tests with Dunnett’s T3 multiple comparisons test with significance accepted at *p* < 0.05, with significant differences denoted by different letters above each column.

**Figure 7:**
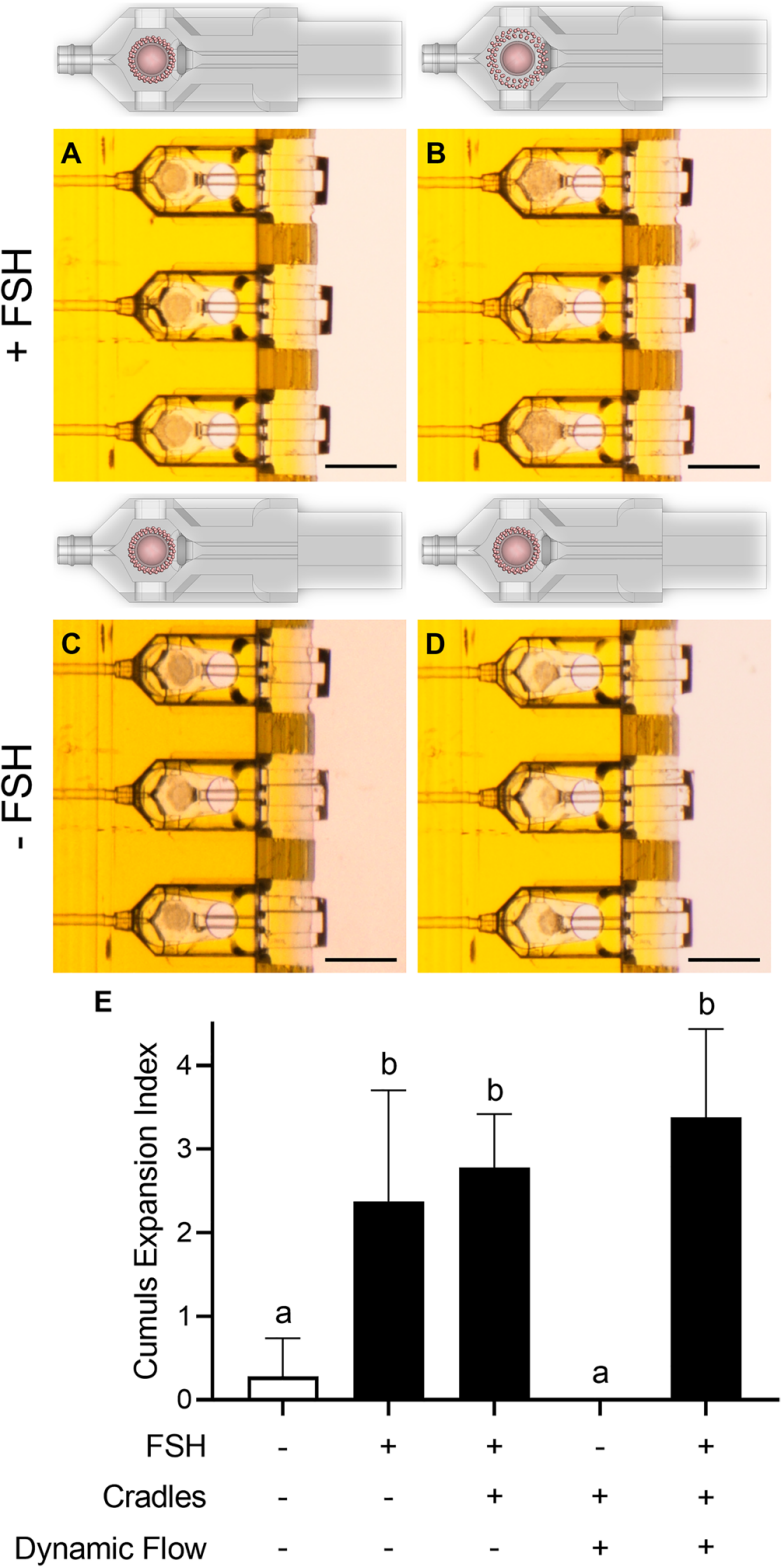
Cumulus expansion in the dynamic flow device in the presence and absence of recombinant human follicle stimulating hormone (rhFSH; Scale bars = 300 µm; 3 reps, n = 8). (**A**, **C**) Mouse cumulus-oocyte-complexes (COCs) before flow begins; (**B**) Mouse COCs after exposure to rhFSH via dynamic flow following 16 h of culture; (**D**) Mouse COCs after no exposure to rhFSH via dynamic flow following 16 h of culture; (**E**) Mean cumulus expansion index (CEI; ± SD) of cumulus expansion following exposure to rhFSH with groups containing cradles housing the COCs in the cradles and dynamic flow groups in flow conditions; Kruskal–Wallis test with Dunn’s multiple comparisons test with significance accepted at *p* < 0.05, with significant differences denoted by different letters above each column.

Two different methods were used to demonstrate flow of biological compounds from the external reservoir of the testbed could reach the cradle and impact cells. For the first method, trypan blue supplemented medium was delivered to Triton-X treated and untreated HEK293 spheroids (Supplementary Video 1). Trypan blue entered cells with damaged membranes around the edge of the spheroids inside the cradle (Fig. 6A–D). Equivalent staining of non-viable cells was observed in spheroids stained in both static and dynamic flow conditions (Fig. 6F–G), with pixel intensity not significantly different between the two methods (Fig. 6H). Unstained spheroid controls exhibited minimal staining (Fig. 6E). For the second method, the delivery of recombinant human Follicle Stimulating Hormone (rhFSH) was assessed through evaluation of mouse cumulus cell expansion (Supplementary Video 2). Expansion was equivalent (*P* > 0.05) between cumulus-oocyte-complexes (COCs) exposed in static culture and those matured in the cradle (Fig. 7E). Both statically and dynamically, the cumulus expansion index (CEI) for COCs exposed to rhFSH was significantly higher than for COCs cultured in the absence of rhFSH (Fig. 7).

### Post compaction mouse embryo development

**Figure 8:**
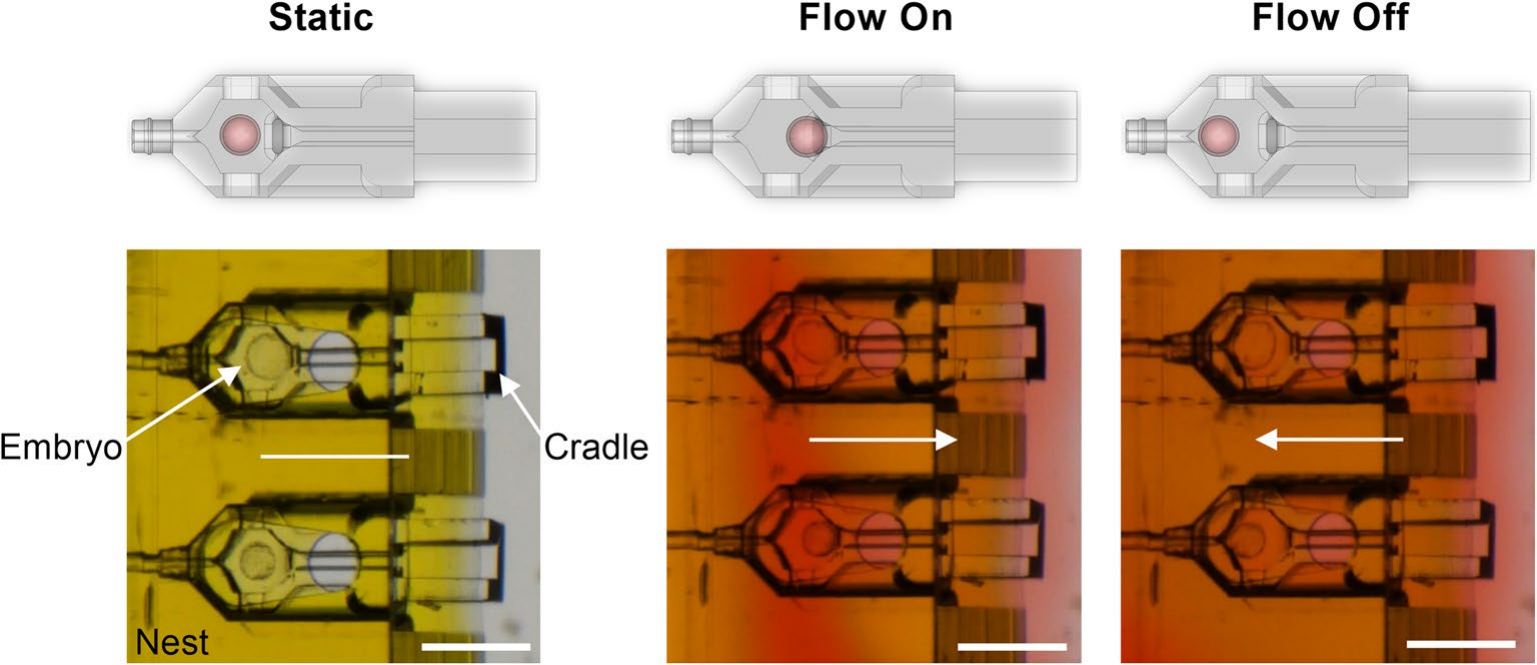
Mouse embryo movement in a changing dynamic flow environment with the introduction of red stained medium (Scale bars = 300 µm; 1 rep, n = 8). (**A**) Static conditions show the expanded blastocysts housed inside the cradles and nest after 48 h of culture; (**B**) Flow On shows the embryos move to the right as the flow pushed them to the back of the cell chamber; (**C**) Flow Off shows the embryos move to the left back towards the nozzle.

Mouse embryo development from the morula to the blastocyst stage was observed under discontinuous flow conditions in the device over a 48-h time lapse imaging period (Supplementary Video 3). While flow was on, embryos were pushed back against the opening of the injection channel. The cell chamber dimensions did not restrict expansion of the blastocyst (Fig. 8; Supplementary Video 3). Repeated cycles of stop-start flow over a duration of 2 min and 40 s, using a subset of the expanded and hatching blastocysts after 48 h of culture, confirmed the embryos move backwards and forwards when the medium flow was turned on or off (Fig. 8; Supplementary Video 4).

## Discussion

To the authors knowledge, this is the first report of a microfluidic chip entirely printed by 2PP with high resolution of multiple features that interfaces reversibly with a second 2PP part to create a unified microfluidic device (Fig. 1). These features included incorporation of medium flow through ten 50 µm diameter channels and insertable cradles with a nozzle that engages with each channel to link medium flow to a cell culture cavity. CFD analysis predictions well reflected the results observed through microbead tracking through the channels. Variation of flow across the channels was predicted and observed, with approximately 30% higher flow in the central channels compared to the outer (Fig. 3B), whereby central channels 3–7 had a high uniformity of velocities (Fig. 5B), with a significant reduction observed in the outer channel 2. Each channel’s mirrored pair produced similar results, so channels 8 and 9, which were not observed in the same recording, were expected to behave similarly to channels 3 and 2 respectively. CFD analysis also successfully predicted flow velocity would accelerate through the nozzle before rapidly decelerating upon entering the cell chamber (Figs. 3A–D, 5A). The final flow rate in the microfluidic channels averaged around 8400 µm/sec (Fig. 5B), with the cradle nozzle flow peaking before slowing the flow further to around 500 µm/sec (Fig. 5C). CFD analysis similarly predicted the spread of flow from the nozzle, with evenly distributed low velocity flow through the cell chamber generating a specific pattern (Fig. 3C), likewise confirmed by microbead experiments (Fig. 3E). Consequently, this study delivers a 2PP device that delivered sub-microliter volume flowrates, together with flow patterns for even distribution of flow through the cell chamber of the cradle. Further, the correlation between CFD analysis predictions and experimental observations, allows for future geometry enhancements to be confidently tested theoretically using CFD modelling.

Specific design elements of the nest and cradle microfluidic device enabled the delivery of sub-microliter flow rates. Firstly, the combination of flow pathways allowed for the inlet flow to be ratioed and divided between the cradles and the outlet (Fig. 1C). As predicted by the CFD (Fig. 3), the lower resistance of the 300 µm outlet redirects the flow, delivering an order of magnitude lower flowrate to the 50 µm nest channels than the piezoelectric pump can deliver. The second key design element was the nozzle barb connection between the cradle and nest. The cradle’s barb has a sealing feature to interlock with the nest channel and port fluid flow through a 30 µm inner diameter to the cell chamber. The feature resolution was only possible with 2PP which, combined with the polymer’s inherent flexibility, enabled cradles to be docked and removed from the nest without breakage or loss of the sealing interface. Reduction of the internal diameter of the nozzle to 30 µm also allowed incoming channel flow to be slowed on entry and evenly dispersed across the chamber’s volume (Fig. 3C–F), providing for even distribution of medium flow in a low shear stress environment. Retention of the redundant cell loading hole in the roof of the nest also pulled flow upwards, further contributing to reducing shear stress on the cells (Fig. 3D, F). These factors counteracted the differences in flow rate observed between the central and outer channels of the nest prior to reaching the cradle. Future iterations of this device can further enhance uniformity by equalising the channel length to each cradle insertion point.

Distinct cellular events relevant to the culture of COCs and preimplantation stage embryos were supported in the nest and cradle microfluidic device. Fluid delivery through the device supported post-compaction mouse pre-implantation embryo development from the morula to the blastocyst stage with medium flow enough to move the embryo within the cradle and pushing it against a pre-formed channel eliminating unstirred layers that occur during static cell culture (Fig. 8, Supplementary Video 3, Supplementary Video 4)^1^. The movement of embryos back towards the nozzle of the cradle when the flow is turned off is a potential demonstration of backflow in this system (Fig. 8). However, further characterisation of this behaviour is required to confirm this, and backflow may be influenced in future systems by the components of the microfluidic layout outside the culture dish and their relative position to each other (shown in Fig. 2A, E). Embryo movement in microfluidic devices has been observed in previous studies that have used fluid flow to move the embryo in order to trap them in specific locations^26^. Future experimentation will need to consider the shear stress placed on the embryo, as it’s known to be detrimental at levels reported at 0.12 Pa^27^. Alternatively, approximately 170-times lower shear stress at 0.7 mPa imparts a positive impact on in vitro embryo development^27^. Because of the ease of removal of embryos, the novel device used in this study is potentially well suited to accurately assessing the impact of shear stress on developmental outcomes in future studies. Although not measured to the authors knowledge, embryos moving through the constraints of the oviduct and uterus under muscular contraction during pre-implantation development in vivo are likely to experience shear forces similar to venous flow^28^, which is similar to the shear stress that has a positive impact on embryo development^29^. Therefore, this system may present an embryo culture model that more accurately reflects the in vivo environment.

Three-dimensional cell culture is a rapidly expanding tool for examining cell–cell interactions, cellular differentiation and for drug efficacy and diagnostic studies, as it better replicates the in vivo environment than 2D-culture^30^. While traditional cell culture platforms aim to replicate dynamic microenvironmental changes, they necessitate interruption and are accompanied by significant swings in nutrient and biological molecule availability. The results demonstrate both chemicals and biological molecules, such as hormones, delivered to spheroids or immature COCs respectively via the 2PP device, elicit appropriate and expected changes to their cellular phenotype. Cumulus cell expansion in vitro is activated by a hormone ligand^31^; in this case by a relatively high concentration of extracellular FSH (Fig. 7, Supplementary Video 2). Cumulus expansion was observed when FSH-containing medium was introduced to COCs by dynamic flow, demonstrating the successful delivery of a hormone through the nest, which triggered an equivalent cellular response to manual intervention (Fig. 7E). Similarly, trypan blue staining was delivered through dynamic flow to pre-established 3D cell spheroids treated with a permeabilization agent to induce compromised membrane integrity (Fig. 6, Supplementary Video 1), with equivalent staining observed between fluidic and manually stained groups (Fig. 6H). Although other microfluidic platforms enable both 2D- and 3D-cell culture, this system was designed to allow retrieval during IVF applications which subsequently allows for cell retrieval after culture (see Fig. 6G). Combined, these data demonstrate the versatility of this system and indicate that the flow generated by this system can support cell growth and division, moving cells, delivering biological molecules, and delivering substances that stain cells within the system, along with removal of cells for downstream applications. Therefore, this delivery system could be used in several other different cell culture applications and cell types.

The 2PP printed nest and cradles were made using proprietary acrylates with the trade names UpPhoto and UpOpto respectively (UpNano GmbH, Vienna, Austria). These polymers are biocompatible and non-cytotoxic after washing with ethanol, consistent with printed structures from other 3D printing methods^32–35^. Additionally, UpOpto is highly transmissive to visible light and suitably stiff to create rigid structures, while still being sufficiently flexible under elastic strain, enabling capabilities such as nozzle insertion within the nest. The use of these materials in 2PP addresses the limitations of PDMS by avoiding leaching that affects cell physiology and viability, absorption of molecules from media and water evaporation^36,37^. Unlike PDMS, it also has the potential to be scaled up for industrial applications as hundreds of parts can be reproducibly printed in a single print job. Importantly, according to the manufacturer’s specifications, precise 3D geometry is achievable with 2PP and allows for printing of features as small as 200 nm in size, enabling specific design elements to be incorporated into the microdevice. Furthermore, with the development of new polymers, 2PP-printed microfluidic chips are set to explore printing with combinations of different polymers that provide both optical features, such as low fluorescence and transparency, and biologically relevant functional features attractive to medical and biological applications. The system presented in this study provides a workable platform to instigate automated media changes during in vitro cell culture due to its equivalent delivery of substances compared to manual handling, with the benefit of cell retrieval following culture. Such innovation is working towards automation of in vitro embryo culture as a method to reduce human variation that occurs between individuals within and between different IVF clinics^38,39^. The move to automation in the IVF sector is led by the necessity to control culture environments, but equally applies to all small batch cell culture applications, including production and investigation of stem cell production and differentiation, spheroid, and organoid cell culture. Control of the environment for precision culture will benefit enormously from the application of automation.

## Conclusion

Printing of a two-part microfluidic device capable of nanoliter volume flowrates of ~ 600 nL/min was accomplished entirely using 2PP. Fluid behaviour within the 2PP device was accurately predicted by CFD, which can be used in future studies to inform prototyping of fluidic devices for specific applications. The 2PP printing provided complex geometry previously unattainable in biologically compatible microfluidic cell culture devices. This nest and cradle device can deliver culture media, dyes and biological molecules to facilitate quantifiable and physiologically relevant alterations in cell growth and morphology while allowing cell retrieval for additional functional analysis. Future development of this technology is ongoing to facilitate advances in automated in vitro embryo culture and three-dimensional cell culture on a microscale.

## Methods

### Chemicals and reagents

Unless otherwise specified, all chemicals and reagents were purchased from Merck-Sigma-Aldrich (St. Louis, MO, USA) and sterile polystyrene cell culture consumables were sourced from Falcon by Corning (New York City, NY, USA).

### Design of the sub-microfluidic platform and CFD modelling

Solidworks 2021 and 2022 were used to design the micro 3D parts of the microfluidic system (Fig. 1; Dassault Systèmes SOLIDWORKS Corp., Waltham, Massachusetts, USA). The cradle was first designed as a support structure for oocytes and embryos to be housed in a cell chamber (denoted ‘e’ in Fig. 1D), which allowed for microinjection through an injection channel (denoted ‘f’ in Fig. 1D). This basic structure can be observed in related previous studies, where the cradle is referred to as a ‘pod’^40,41^. Two distinct features were added to the cradle in this study; the fin (denoted ‘g’ in Fig. 1D) to provide a larger structure to hold with fine forceps to aid in cradle removal from the nest, and the barbed nozzle (denoted ‘d’ in Fig. 1D) to enable reversible bonding between the cradle and the nest. Cell manipulation was achieved through an opening at the top of the cradle cell chamber (Fig. 1B, denoted ‘e’ in D) whilst the nest acted as a roof once the cradle was inserted. A hole was present at the back of the roof that sits above each inserted cradle (Fig. 2B) so the cradle could be partially inserted, cells could be loaded, and the cradle could be fully docked into the nest. However, it was easier to load cells with the cradle outside of the nest once testing began. It remained as a feature in the nest’s design when CFD predicted it allowed fluid to escape upwards, reducing the amount of flow directed towards the injection channel (Fig. 3D). The nest was designed to be a link between the cradle and the fluidic system, with the cradles inserting into a complimentary recess 300 µm across while also connecting to tubing with an internal diameter of 1.3 mm. This enabled fluid to be delivered from a macroscale fluidics system to a microscale cell culture support structure (see Fig. 2A, D, C, B). Once the design had been modelled, the design file could be used for CFD analysis.

Solidworks Flow Simulation 2021 and Solidworks Flow Simulation 2022 (Dassault Systèmes SOLIDWORKS Corp.) were used as the CFD modelling software to simulate fluid flow through and around parts to understand resulting flows, stresses, thermal changes and mixing. The parameters for the CFD analysis tested water under laminar flow (Reynolds Number ~ 0.3) at 37 °C with 9.81 m/s^2^ gravity and a channel wall roughness of 1 µm, the approximate accuracy of 2PP printing. A critical factor in the success of CFD simulation is the design of the mesh. Meshing, also known as mesh generation, is the process of generating a two-dimensional and three-dimensional grid, dividing complex geometries into elements that can be used to discretize a domain. In this case, the mesh was optimized to provide high resolution for each individual nest and cradle (approximately 6 cells across each 50 µm diameter channel and 8 cells across the 260 µm width of the cell chamber). Inlet and outlet flows were inserted as boundary conditions into the model per preliminary mass-based experimental flowrate estimates. The CFD simulation was initially validated against the results of particle tracking velocimetry by comparing it with CFD particle studies using simulated polystyrene particles of the same dimensions. Once the model produced a qualitatively similar result as determined by a visual inspection, boundary conditions were iterated to achieve the mean channel flow rates demonstrated experimentally with particle tracking velocimetry. A cut plot of velocity indicated 1 × 10^–2^ m/s was a suitable cutoff for a ‘high’ velocity.

### Printing and washing of the sub-microfluidic platform

Cradles and the nest were 2PP printed on a NanoOne high-resolution 3D printer from proprietary biocompatible acrylate-based 2PP resins with the trade names UpOpto and UpPhoto respectively (UpNano GmbH, Vienna, Austria). Following modelling of the 3D parts in Solidworks (Fig. 1), resulting STL files were imported into Think3D software (UpNano GmbH). Cradles were printed in vat mode using a 20 × objective (numerical aperture 0.7, printing resolution in XY is 420 nm and in Z is 2.75 µm) with a water immersion medium and a transparent low fluorescent resin (UpOpto). An adapted print profile was used, delivering a laser power setting of 150 mW to match the objective-material combination. Coarse printing mode was enabled for the optimal balance between printing resolution and speed, achieving a print time of 7 h and 53 min for an array of 196 parts. The nest was printed in vat mode using a 10 × objective (numerical aperture 0.4, printing resolution in XY is 730 nm and in Z is 8.81 µm) with an air immersion medium and UpNano’s high performance polymer (UpPhoto). Predefined individual print profiles were used, delivering a laser power setting ranging from 50 to 100 mW to match the objective-material combination. Adaptive resolution printing mode was enabled for optimal balance between printing resolution and speed, allowing for a majority of the nest to be printed in course mode before switching to fine mode to print the details of the cradle insertion points (Fig. 1E), achieving a print time of 4 h and 31 min for one complete fully cured part.

Both cradles and the nest were post processed and developed by sequential immersion in three consecutive isopropanol baths. For the nest, further post processing was performed by flushing internal 50 µm channels with isopropanol using a pressure-controlled pump up to 3 bar. For the cradles, a post curing step of UV light at 365 nm for ~ 1 h was required. After post processing, the nest was washed in 3 mL of 96% ethanol (Chem-Supply Pty Ltd, Gillman, SA, Australia) and rinsed twice in 6 mL of 5% 7X detergent (MP Biomedicals Australasia, Seven Hills, NSW, Australia), using a P200 micropipette to draw fluid through the nest channels, cradle insertion points and inlet and outlet channels (Fig. 1A, C) prior to soaking in 7X overnight. The nest was then rinsed three times in 10 mL of phosphate buffered saline (PBS) and soaked in cold PBS (4–8 °C) for two days, before storage in fresh cold PBS until each experiment was conducted. In contrast, cradles were cleaned using sonication (Elma Ultrasonic Bath, Techspan Australia PTY Limited, NSW, Australia) for 20 min at 40 °C in 96% ethanol and 5% 7X detergent, with two rinse steps in between the two sonication steps. After sonicating in 7X, the cradles were rinsed twice in 2 mL of cold PBS before being stored in 3 mL of cold PBS. Washing preceded all experimentation described below.

### Testbed setup

All materials for the testbed (Fig. 2) were purchased from Elveflow (Paris, France), Darwin Microfluidics (Paris, France) or Bartels Mikrotechnik (Dortmund, Germany) and selected based upon known biocompatibility, sterilizability and availability. Microfluidic tubing connecting the components of the testbed was either 1.3 mm inner diameter (ID) Tygon® tubing (mp-t; Bartels Mikrotechnik) or 1/16″ outer diameter (OD) 1/32″ ID polytetrafluoroethylene (PTFE) tubing (Elveflow) accommodating variation in connector types amongst components used in the system. Joins were made using Idex Flangeless Teflon™ fittings with perfluoroalkoxyTefzel™-ethylene tetrafluoroethylene (ETFE) ferrules 1/4″-28 on 1/16″ OD PTFE tubing coupled with 1/4″-28 Tefzel™-ETFE union joins (Elveflow), or by sheathing the 1/16″ OD tubing inside the 1.3 mm ID tubing (Fig. 2). Y-joins were made using polypropylene connectors (mp-y, Bartels Mikrotechnik). An external fluid reservoir and manual injection port were connected in line and able to be isolated via manual shutoff valves (0.51 mm ID, Tefzel™-ETFE, Elveflow). Flow was driven by a piezoelectric pump (mp6-liq, Bartels Mikrotechnik) with backflow prevented by a check valve (mp-cv, Bartels Mikrotechnik). Flow was regulated by the insertion of 75 mm Idex polyether ether-ketone (PEEK) capillary tubing of 250 μm ID (Darwin Microfluidics). The main fluid line can be directed to the nest or purged to a waste reservoir using electronically switchable shape-memory alloy valves (SMV-2R-BN1F, Takasago Electric, Inc., Nagoya, Japan). The outlet was left to drain freely into an open container to reduce back pressure to the nest. The piezoelectric pump and electronic valves were operated using a mp-Multiboard evaluation board equipped with mp-Highdriver4 and mp-valvedriver, combined with an Arduino microcontroller to drive the pumps. Control software (version 1.69) connected to the board over a USB interface, while direct DC power was independently supplied to the board.

The nest was held flat in a 60 mm petri-dish with a custom designed 35 mm titanium 3D printed block (ProX DMP200 Printer, 3D Systems Inc., Rock Hill, SC, USA), with 50 µm tolerance, allowing space for fluid to fill the dish over the course of an experiment. For biological experiments, the testbed and components were mounted on a heating stage and protected from external light. Imaging was achieved using a Nikon SMZ18 Stereomicroscope equipped with a Nikon DS-Fi3 camera (Nikon Corporation, Tokyo, Japan). Channels of the nest were primed with 70% ethanol, 5% 7X, 70% ethanol, Milli-Q Water (Merck Millipore Ltd, MA, USA) and finally culture medium for all biological experiments. The external surface of the manifold was rinsed with 7X to remove any paraffin oil (Merk Group, Darmstadt, Germany), ethanol for sterilisation, Milli-Q Water to remove any residual ethanol and finally submerged in a culture medium bath.

### Particle tracking velocimetry (PTV)

For CFD validation, the fluidic system was modified to incorporate a second pump line to allow for injection of microbeads close to the nest (Supplementary Fig. 1) to track the flow rate of particles carried through the fluid channels. Polystyrene particles with a diameter distribution of 6.7 ± 0.9 µm (n > 3200 particles; F1CP-50-2 nominal diameter 5 µm, Spherotech, IL, USA) were diluted to 0.025% w/v in H_2_O and mixed with red food dye (1:20) to allow visualization of the probe front. Flow was driven by 25 V pulses to the piezoelectric pump at 75 Hz, with particles injected into the system with the second pump in pulses, using the same pump settings, while the valve to the injection line was closed. The system was enclosed in an OkoLab Stagetop Incubator set at 37 °C (OKOLAB USA Inc., Ambridge, PA, USA) mounted on an Olympus IX83 inverted microscope (Microscopy Technologies, Olympus Corporation, Tokyo, Japan) equipped with a Teledyne Prime BSI sCMOS camera (Teledyne Photometrics, Tucson, AZ, USA). Videos were recorded using 4 × or 10 × APO objectives at 62.5 (2048 × 2048) or 93 fps (reduced frame) and particle velocities were measured using the frame-to-frame distance travelled by individual microbeads. The maximum field of view at adequate magnification to track the particles could image 6 channels at a time so channels 2–7 were recorded. Particle velocities were converted to flow rates using the cross-sectional area of the 50 µm diameter channel (1963.5 µm^2^).

### Biological outcome testing

All biological experiments were captured with either video or timelapse imaging so changes could be assessed by multiple researchers to avoid bias as treatment groups could not be blinded.

#### Laboratory animals

CBA x C57Bl/6 F1 hybrid mice were obtained from Laboratory Animal Services (LAS; University of Adelaide, SA, Australia) and were housed under controlled temperature and maintained on a 12 h light: 12 h dark cycle with ad libitum rodent chow and water. To stimulate follicle growth, pre-pubertal female CBA x C57Bl/6 F1 hybrid mice (21–24 days old; 8.5–10 g) were administered 5 IU equine chorionic gonadotrophin (eCG; Braeside, VIC, Australia) via intra-peritoneal injection. For embryo collection, female mice were subsequently injected 46 h later with 5 IU human chorionic gonadotrophin (hCG; Kilsyth, VIC, Australia) to induce ovulation and mated overnight with CBA x C57Bl/6 F1 hybrid male mice (6–8 weeks old; 26–31 g). Female mice were humanely culled by cervical dislocation at either 46 h post eCG administration for the collection of immature cumulus-oocyte complexes (COCs; n = 6 female mice) or 22 h post hCG and mating (n = 1 male mouse) for the collection of pronucleate oocytes (2PN; n = 1 female mouse). Any reproductive tracts collected post mating with no sperm surrounding the 2PNs were excluded. Male mice were humanely culled by cervical dislocation at the conclusion of the study.

#### Cumulus-oocyte-complex (COC) collection

Mouse ovaries were collected in Research Wash medium (ART Lab Solutions, SA, Australia) supplemented with 4 mg/mL fatty acid free bovine serum albumin (BSA; MP Biomedicals, AlbumiNZ, Auckland, New Zealand). Immature COCs were isolated by puncturing preovulatory follicles on the ovary using a 29- gauge needle (Terumo Australia Pty Ltd, NSW, Australia) in α–MEM handling medium (Gibco, Thermo Fisher Scientific, Waltham, MA, USA) lacking deoxyribonucleosides, D-glucose, L-glutamine, ribonucleosides or sodium bicarbonate and supplemented with 5.95 mM sodium bicarbonate, 10 mM HEPES (acid), 10 mM HEPES sodium salt, 5.55 mM D-(+)-glucose, 0.05 g/L of gentamicin sulfate, 10 mL of 100X GlutaMAX (Gibco), 3 mg/mL BSA (MP Biomedicals) and 1 mg/mL Fetuin before being randomly allocated into treatment groups.

#### Cumulus cell matrix expansion

Immature COCs were cultured for 17 h in α-MEM handling medium in the presence or absence of 50 mIU/mL recombinant human Follicle Stimulating Hormone (rhFSH; Los Angeles Biomedical Research Institute, LA, USA) under static or dynamic flow culture conditions at 37 °C in air. Static conditions consisted of 20 µL drops (1 COC/2 µl) in 60 mm petri dishes under paraffin oil (Merk Group) cultured both within cradles and nests and alone in the presence and absence of rhFSH in an ungassed incubator. For dynamic flow culture conditions, COCs were transferred to a 60 mm petri dish containing cradles and the nest bathed in α-MEM handling medium (± rhFSH). COCs were loaded into cradles with cradles via the open cell chamber ‘e’ (Fig. 1D) prior to being docked into the nest using Dumont Dumostar fine forceps (0.025 mm × 0.015 mm tips, Coherent Scientific, Thebarton, SA, Australia) and before flow was initiated. Continuous flow of α-MEM handling medium (± rhFSH) was run with 25 V pulses to the piezoelectric pump at 75, corresponding to a channel flow rate of ~ 600 nL per minute (Supplementary Fig. 2), for 1 h followed by 16 h of static culture on the stereomicroscope with timelapse images captured every 5 min. After 17 h of culture, COCs were removed from drops or the nest and cumulus cell expansion was scored using an Olympus CK2 inverted microscope (Olympus, Tokyo, Japan) according to the well established cumulus expansion scoring index as defined by Vanderhyden et al.^42^ For COCs inside cradles and the nest, they were scored both inside cradles and then outside the cradles after they had been retrieved. The score taken outside the cradle was used for statistical analysis unless the cumulus cells were stripped when the COC was removed from the cradle and the cumulus expansion could no longer be scored.

#### Pronucleate oocyte (2PN) collection

Mouse oviducts were collected in Research Wash medium (ART Lab Solutions) supplemented with 4 mg/mL BSA (MP Biomedicals). Pronucleate oocytes (2PNs) were liberated from the ampulla using a 29-gauge needle (Terumo Australia Pty Ltd). Viable 2PNs were collected using a flame pulled Kimble 9″ Soda Lime Pasteur Pipette (Bio-Strategy Pty Ltd, Tullamarine, VIC, Australia) and briefly treated with 50 U/mL hyaluronidase diluted in Research Wash medium to remove cumulus cells. Once denuded, 2PNs were washed twice in Research Wash medium then randomly allocated into treatment groups.

#### Post-compaction embryo culture

2PNs were cultured overnight in 20 µL drops (1 per 2 µL) of Research Cleave Media (ART Lab Solutions) supplemented with 4 mg/mL BSA (MP Biomedicals) overlaid with paraffin oil (Merk Group) at 37 °C in humidified air comprising 5% O_2_, 6% CO_2_, 89% N_2_. Fertilization was scored 24 h after 2PN collection with cleaved embryos moved to a fresh culture drop until 72 h post 2PN collection. Resultant morula and early blastocysts were transferred from standard culture conditions into a 60 mm petri dish containing the nest and ~ 8 mL of Research Wash medium, then loaded into individual cradles via the cell chamber opening using a flame-pulled Pasteur pipette (Bio-Strategy Pty Ltd). Using fine forceps, the cradles were docked into each individual nest channel prior to culture. Time-lapse imaging was initiated and run for 24 h at 37 °C on the stereomicroscope, capturing images every 5 min. Discontinuous flow through the nest was initiated with 25 V pulses at 75 Hz, corresponding to a flow rate of ~ 600 nL per minute (Supplementary Fig. 2). At 96 h after 2PN collection, embryo development was assessed within the cradle. Flow testing with the expanded blastocysts was undertaken using handling medium with red food dye (1:20 dilution) at room temperature. In repetitious cycles, the flow was turned on and off to determine how the presence of the flow influenced embryo positioning within the cell chamber of the cradle.

#### HEK293 cell culture

Human Embryonic Kidney 293 cells (HEK293; Australian Biosearch, Wangara, WA, Australia) were cultured in Dulbecco’s Minimal Essential Medium (DMEM, Gibco) supplemented with 50 U/mL Penicillin G, 50 µg/mL streptomycin, 2 mM L-Glutamine, 0.1 mM MEM Non Essential Amino Acids (MEM NEAA; Gibco) and 10% (v/v) Foetal Bovine Serum (FBS; Thermo Fisher Scientific) (DMEM + FBS) under standard monolayer cell culture conditions (37 °C, 5% CO_2_ in air, 95% humidity) in a Nunc T25 flask (Thermo Fisher Scientific). Following routine subculture, confluent HEK293 cells were used to create spheroids. Adherent cells were trypsinized (0.25% trypsin–EDTA, Gibco), with trypsin neutralized by addition of DMEM + FBS. The cell suspension was centrifuged at 200 g for 5 min. Cells were then resuspended in 5 mL of DMEM + FBS and repeatedly pipetted to form a single cell suspension. Cells were diluted with DMEM + FBS to a concentration of 1.25 × 10^4^ cells/mL. Hanging drop cultures were prepared using 5 µL of cell suspension added to 15 µL drops positioned on the underside of 60 mm petri dishes lids. Lids were gently swirled to encourage cell aggregation before inversion over the petri dish bases, each containing 7 mL sterile H_2_O. Dishes were incubated at 37 °C in a humidified atmosphere of 5% CO_2_ in air.

#### Trypan blue staining

HEK293 spheroids were removed from hanging drops using a flame-pulled pasteur pipette (Bio-Strategy Pty Ltd) and transferred to one well of a Nunc 4 well dish (4WD; Thermo Fisher) containing 750 µL of HEPES-buffered DMEM (Gibco) supplemented with 50 U/mL Penicillin G, 50 µg/mL streptomycin, 2 mM L-Glutamine, 0.1 mM MEM NEAA (Gibco), 1 mM sodium pyruvate and 0.3 mg/mL Polyvinyl alcohol (PVA) (H-DMEM + PVA). Spheroids were briefly washed and then either partially permeabilized with 0.05% (v/v) Triton X-100 (USBiological, Salem, MA, USA) for 30 secs at room temperature to facilitate the uptake of trypan blue (Thermo Fisher) or left untreated. Triton X-100 treated spheroids were washed through two 750 µL washes of H-DMEM + PVA and then randomly allocated to staining under either static or fluidic conditions. Static staining was performed by incubating spheroids in 0.02% trypan blue for 5 min at room temperature in a 4WD followed by two H-DMEM + PVA washes. Unstained control spheroids were generated by incubating in H-DMEM + PVA without trypan blue. Triton X-100 treated spheroids allocated to fluidic staining were loaded into cradles using a fine-pulled glass pipette which were then docked into individual nests within the microfluidic device prior to the initiation of medium flow as described above. Flow was video captured at 30 fps on the stereomicroscope and run as per the late embryo culture experiment for 7 min before medium was switched to H-DMEM + PVA. Flow was stopped after ~ 20 min, when the medium in the nests was observed to be clear of trypan blue, at which point spheroids were removed and placed into fresh- DMEM + PVA. Spheroids were retrieved by undocking each cradle from the nest using fine forceps followed by removal using a fine-pulled glass pipette. All individual spheroids were imaged on the stereomicroscope after the flow experiment was complete.

### Image analysis

Image Analysis was performed using Fiji Is Just ImageJ (FIJI) freeware^43^. Microbeads were tracked manually in image stacks using the multipoint and region of interest (ROI) multimeasure tool. Timelapse data for the post compaction embryo development and COC culture were stabilized using scale invariant feature transformation (SIFT) registration. Microbeads were tracked manually in image stacks using the multipoint and region of interest (ROI) multimeasure tools. For trypan blue stained spheroid image analysis, images were converted to 8-bit greyscale then the perimeter of each individual spheroid was traced to determine mean pixel intensity in each ROI. The mean pixel intensity was transformed to reflect pixel darkness by subtracting the measured value from 255, the maximum pixel intensity value. Mean pixel darkness was then compared between spheroids in different treatment groups.

### Statistical analysis

Biological outcomes were analysed using GraphPad Prism 9 software (GraphPad Software, San Diego, CA, USA). All data were analysed for normality using the Kolmogorov–Smirnov test and homoscedasticity by assessing a simple linear regression residual plot. For trypan blue image analysis, mean pixel darkness was compared using Brown-Forsythe and Welch’s ANOVA tests with Dunnett’s T3 multiple comparisons test. Cumulus expansion index (CEI) scores for each treatment group were compared using a non-parametric Kruskal–Wallis test with Dunn’s multiple comparisons test. Significance was accepted at *p* < 0.05. All data are presented as mean ± SD.

### Ethics declaration

All animal experiments in this study were approved by The University of Adelaide Animal Ethics Committee (M-2021-052) and were conducted in accordance with the Australian Code of Practice for the Care and Use of Animals for Scientific Purposes. The results of all animal experiments in this study are reported in accordance with ARRIVE guidelines.

## Supplementary Information

Supplementary Video 1.

Supplementary Video 2.

Supplementary Video 3.

Supplementary Video 4.

Supplementary Information 1.

## Data Availability

The data that support the findings of this study are available from Fertilis Pty Ltd, but restrictions apply to the availability of these data and are not publicly available. Data are however available from the corresponding authors upon reasonable request and with permission of Fertilis Pty Ltd.
