# Supplementary material for: Nano-liter perfusion microfluidic device made entirely by two-photon polymerization for dynamic cell culture with easy cell recovery
Source: Sci Rep. 2023 Jan 11;13:562. doi: 10.1038/s41598-023-27660-x (PMC9834384; doi:10.1038/s41598-023-27660-x)
Supplement: Supplementary file 5 — Supplementary Information 1. [file 41598_2023_27660_MOESM5_ESM.pdf]

TITLE: Nano-liter perfusion microfluidic device made entirely by two-photon polymerization for dynamic cell culture with easy cell recovery.

AUTHORS: Hanna. J. McLennan[1], Adam J. Blanch[1], Samuel J. Wallace[2], Lesley J. Ritter[1, 3], Shauna L. Heinrich[1], David K. Gardner[1, 4, 5], Kylie R. Dunning[3, 6, 7, 8], Marty J. Gauvin[1, 2, 9], Allison K. Love[1], Jeremy. G. Thompson[1, 6, 10].

DOI: 10.1038/s41598-023-27660-x

Supplementary Information

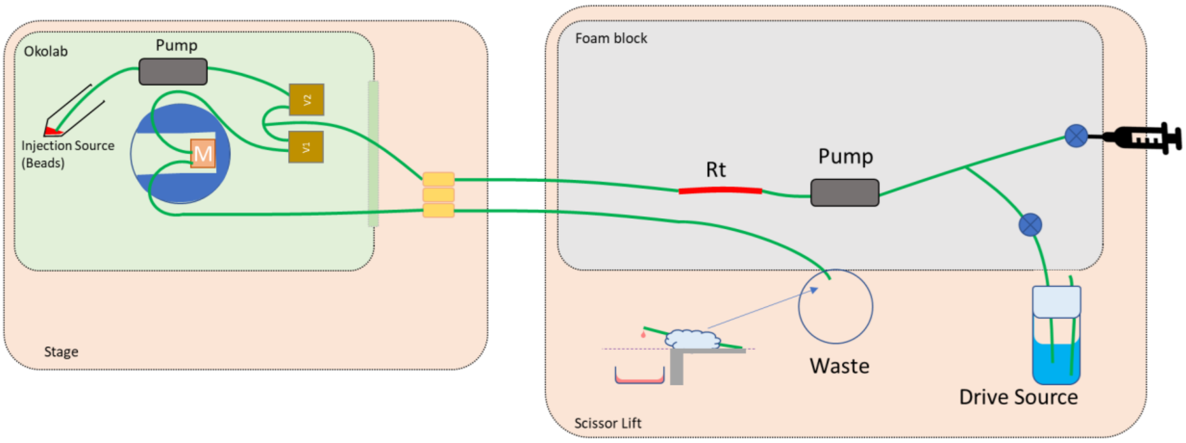

Supplementary Figure 1: Testbed layout for microbead tracking experiments

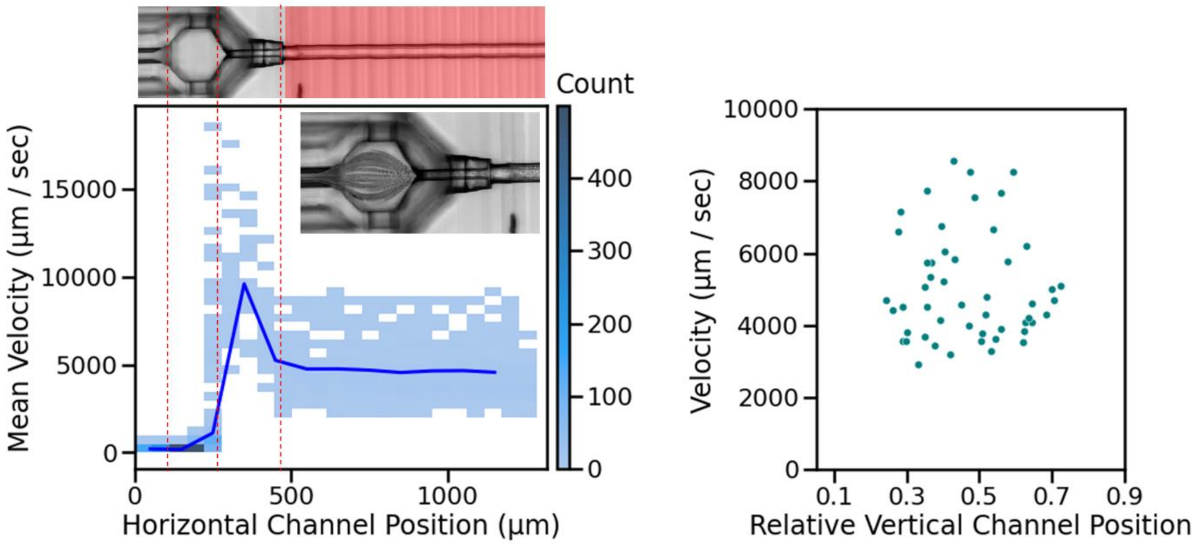

Supplementary Figure 2: 2D histograms (left) of bead velocity per channel length for channel #7, when the total system flow (outlet + channels) was ca. 60.2 µL/min. The blue trace is the mean velocity profile, with the corresponding projection of bead tracks (inset). Red shading indicates the region over which the mean channel flow rate was calculated (5022 ± 1487 µm/sec, which in a 50 µm diameter channel is equivalent to 0.59 ± 0.18 µL/min; n=51 particles). Imaging was performed with a 10x objective at 93fps. Velocity profiles within the channel relative to the channel walls are shown (right).

Supplementary Video Legends

Supplementary Video 1: HEK293 cell spheroids treated with trypan blue using dynamic flow delivery

Supplementary Video 2: Mouse cumulus-oocyte-complex expansion in the presence and absence of recombinant human follicle stimulating hormone

Supplementary Video 3: Embryo development from morula to blastocyst

Supplementary Video 4: Expanded mouse blastocysts moving in cradles when medium flow is turned on and off

All supplementary videos are compatible with VLC Media Player. In the event of any playback issues, please contact the corresponding authors.
